# Reclaiming voices, rethinking change: a decolonial and knowledge-ecological analysis of SBCC nutrition interventions in Senegal

**DOI:** 10.3389/fnut.2025.1609237

**Published:** 2025-08-13

**Authors:** Sylvain L. Faye, Georgette H. Sow

**Affiliations:** ^1^Department of Sociology, Cheikh Anta Diop University, Dakar, Senegal; ^2^S&F Pro Consulting, Manteca, CA, United States

**Keywords:** social and behavior change communication, decolonial approaches, nutrition intervention, indigenous knowledge, community engagement, epistemic justice, Senegal

## Abstract

**Introduction:**

Social and Behavior Change Communication (SBCC) strategies have become central to nutrition interventions in Senegal, particularly to combat malnutrition and food insecurity among caregivers. However, improved nutritional knowledge has not consistently led to sustainable dietary practices. This study explores the limitations of conventional SBCC approaches through a decolonial and ecological lens of knowledge.

**Methods:**

This qualitative study draws on ethnographic and participatory research conducted between 2020 and 2024 in Senegal. It focused on malnutrition, stunting, and food fortification initiatives—particularly those involving rice and broth cubes—to investigate the alignment between SBCC messaging and local food cultures.

**Results:**

Findings reveal that prevailing SBCC models often rely on top-down, biomedical messaging shaped by Western nutrition science and state dietary norms. These interventions frequently overlook local food ecologies, sociocultural practices, and community knowledge, reinforcing technocratic and neoliberal framings while limiting community agency.

**Discussion:**

To enhance relevance and sustainability, we advocate for a decolonial and knowledge-ecological approach to SBCC. This includes centering epistemic justice, fostering relational ethics, and co-creating interventions with communities. Integrating local perspectives and plural knowledges can better address the complex socio-ecological drivers of malnutrition.

## Introduction

Many countries worldwide share common challenges related to food insecurity and achieving sustainable, high-quality nutrition. Senegal, a sub-Saharan African country, has been recognized as a low-income exemplar country in nutrition, having made significant progress in reducing child stunting prevalence by 17.9% (from 1992 to 2017) (1) and maintaining the lowest burden of stunting among its neighboring countries. However, inequalities in the prevalence of stunting have persisted across wealth quintiles, maternal levels of education, and areas of residence (2).

The Senegalese government’s top priority for the past few years has been improving the national nutritional situation. International commitments led to the establishment of the Malnutrition Control Unit (CLM) in 2001, which was later renamed the *National Council for Nutrition Development* (CNDN) in 2020. The CNDN aims to support the national strategy for food security and resilience (2015–2035), seeking to increase food availability, improve access to diverse and healthy foods, enhance nutritional status, and increase resilience against climate shocks (3). The Multisectoral Strategic Nutrition (PSMN) 2018–2021 aimed to address malnutrition, focusing on expanding and improving nutrition services, including micronutrient supplementation and food fortification, as well as income-generation cash transfer schemes and opportunities to enhance purchasing power. Additionally, it utilized communication technologies to promote nutrition-specific and health practices. Within the Emerging Senegal Plan (PSE) (4), food security policies to combat malnutrition emphasized strengthening communities’ resilience to shocks with social protection and family safety nets.

Despite these government efforts over the past few decades, Senegal remains food insecure, particularly vulnerable to climate shocks, unsustainable farming systems, and limited market access. The agricultural system’s crisis (2018–2022) has impacted the ability to provide food security for citizens, despite improvements in supply, affordability, and physical proximity. As of March *2022*, Senegal reported a 49.8% prevalence of moderate to severe food insecurity, with 5% of the population experiencing acute *food insecurity* that requires immediate assistance (5). In 2023, government efforts to lower the prices of staple foods have reduced household food insecurity (compared to 2022); nevertheless, 1.3 million people faced acute food insecurity during the lean season, while 4.5% of households reported experiencing hunger. Climate change and extreme weather events have impacted food production through frequent heatwaves, droughts, and flooding, leading to rural population movements and influencing food availability (6). The COVID-19 pandemic has disrupted the food and nutrition supply chain, bringing food sovereignty to the fore (7). The war in Ukraine, poor harvests, and regional instability have significantly increased food and transport costs, impacting global food security and economies, particularly in developing countries (8).

Senegal is not currently on track to meet all Sustainable Development Goal SDG 2.2 targets for maternal, infant, and young child nutrition. Despite progress made in reducing chronic malnutrition in 2023, stunting still affects 18% of children under five. Progress in reducing wasting has been reversed over the last 5 years, with the global acute malnutrition rate among children increasing from 8 to 10% (9). Widespread micronutrient deficiencies remain a public health problem: anemia affects 68% of children under 5 and 53% of women and girls of childbearing age. Food insecurity has forced 22% of households to adopt coping strategies (10), such as reducing health expenditures, the quantity of food consumed, the number of meals taken by adults in favor of children, as well as withdrawing children from school (11). As a result, Senegal ranks 66th out of 119 countries in the Global Hunger Index (12).

Considering that early childhood feeding practices are fundamental for a child’s healthy growth, The World Health Organization (WHO) and UNICEF have established a global strategy for optimal Infant and Young Child Feeding (IYCF) (13), including exclusive breastfeeding (EB) for the first 6 months of life, introducing nutritionally adequate and safe complementary foods at 6 months (14). However, in 2023, 38% of Senegalese children aged 0–23 months were breastfed within an hour of birth. The percentage of exclusively breastfed children increased from 34% (2005) to 41% (2019), then decreased to 34% in 2023. Globally, 10% of Senegalese children under 2 received the minimum acceptable dietary intake and were fed according to IYCF recommendations (15).

Nutrition behavior change programs have been promoted to address inappropriate feeding and caregiving practices and improve child health and nutritional status (16). Optimal complementary feeding has been prioritized through dietary diversification, timely introduction, and the provision of appropriate types and amounts of complementary foods (17). Nutrition education encompasses a range of educational strategies, supplemented by environmental supports, aimed at promoting the voluntary adoption of food choices and other nutrition-related behaviors that promote health and well-being (18). This definition insists on behavior change, communication, and counseling to address participants’ automatic and reflective motivation (19). SBCC has been increasingly used with nutrition-sensitive strategies (agriculture, social safety net, and WASH interventions) to improve nutritional status (20).

Senegal’s Nutrition Reinforcement Program (PRN 2007–2011) focused on a participatory and multisectoral approach (21), introducing an extensive network of volunteer health workers. It conducted monthly screening to identify and treat malnutrition in young children, and raised awareness of exclusive breastfeeding and the importance of a nutritious diet (22). Community involvement was also encouraged within the Learning, Nutritional Rehabilitation, and Awakening Homes (LNRH) program, also known as FARNE. This strategy utilized mothers of well-nourished children, known as positive deviant mothers, and transitioned from nutrition education sessions and cooking demonstrations to self-discovery activities for mothers (23).

However, although parental knowledge of nutritional standards has improved, it does not always induce changes in children’s nutrition and hygiene behavior (24). A study conducted in the Matam region found that, although 65.3% of mothers intended to provide iron-rich foods (IRF) to their children, this intention did not correlate significantly with actual provision. Factors such as the child’s age and household food insecurity were more predictive of IRF consumption than maternal psychosocial factors. A recent study conducted in Dakar highlights the persistent gap between parental knowledge of child nutrition and actual feeding and hygiene practices (25). While most parents, particularly mothers, demonstrated adequate awareness of nutritional standards, several structural and contextual barriers prevent this knowledge from translating into consistent behavior. Key obstacles include economic constraints that limit access to nutritious foods, time poverty among mothers, persistent social norms that conflict with modern dietary recommendations, and inadequate access to safe water and sanitation. The study concludes that improving knowledge alone is insufficient; effective interventions must also address underlying socioeconomic and infrastructural conditions to support sustainable behavior change in child nutrition and hygiene. Most policies implemented in nutrition, health, food security, and social protection highlight inappropriate IYCF, inadequate healthcare and services, and poor-quality diets (26).

How can we understand such a situation in a country that has distinguished itself through community support and communication approaches to promote social and behavioral change? Why have these interventions failed to create an environment conducive to good community feeding practices?

This study does not aim to measure the direct impact of existing SBCC interventions on nutritional outcomes, such as changes in the prevalence of anemia or stunting following fortification programs. Rather than conducting an outcome evaluation, the analysis focuses on the conceptual, ethical, and sociocultural dimensions of how SBCC strategies are designed, communicated, and received. The objective is to critically examine the underlying assumptions, participatory processes, and acceptability of these interventions within their local contexts. While impact indicators, such as micronutrient status or anthropometric measures, are essential for assessing effectiveness, this paper argues that such metrics alone are insufficient for understanding why behavior change occurs or does not. Instead, it highlights the importance of addressing the structural, epistemological, and relational factors that mediate community responses to public health messages.

Although financial resources and food security have long been recognized as determinants of an individual’s dietary intake, the lack of integration of local culture has been a persistent problem in Global Health Research (27). Social norms and cultural factors are increasingly considered in health and nutrition SBCC interventions in Low-and Middle-Income Countries (LMICs) (28). Social norms are rules of behavior that individuals prefer to conform to, provided they believe a sufficient number of people in their reference network conform to them, and that an adequate number of people believe they ought to conform to them (29). Nutritional social norms are the unwritten social rules that influence dietary practices by shaping perceptions of what others do and what others think is appropriate, which can affect individual food choices and child feeding behavior, even when nutritional knowledge is adequate (30). In some Senegalese communities, social norms may discourage young women from eating eggs during pregnancy due to beliefs about childbirth complications, even when mothers know eggs are nutritious. Similarly, exclusive breastfeeding may be hindered if the norm favors the early introduction of water. As informal, shared rules that guide behavior within a group, they include both what individuals perceive others typically do (descriptive norms) and what they believe others approve or disapprove of (injunctive norms). This dual influence makes social norms particularly powerful in shaping health-related behaviors, including nutrition and hygiene practices. SBCC Interventions should be sensitive to social norms, supporting the empowerment of key change agents that can influence other behaviors and facilitate changes toward nutrition determinants (31). However, a gap in nutrition programs in LMIC countries is the lack of responsiveness and systematic attention to their influence (32). Only 6% of World Bank nutrition interventions address social norms, highlighting a significant gap (33). Effective nutrition promotion requires actively hearing and incorporating the voices, perspectives, and needs of the populations concerned to ensure culturally appropriate and sustainable behavior change. Research has shown that interventions designed without community input often fail to resonate with local beliefs, traditions, and socioeconomic realities, limiting their effectiveness (34). For example, in rural West African settings, complementary feeding practices improved significantly when programs engaged caregivers and community leaders to align recommendations with existing cultural norms. Moreover, understanding social norms and the lived experiences of adolescents and families is crucial to designing nutrition strategies that are not only acceptable but also feasible within local contexts (35). Promoting behavior change requires engaging with the target population, understanding their motivation, and adapting interventions to contexts that facilitate change (36). This participatory approach fosters ownership, enhances trust, and ultimately promotes more sustained changes in dietary behaviors.

SBCC Interventions used to improve IYCF practices have shown promising results (37). However, community adherence to promoted nutritional practices still needs to catch up (25). One of the reasons is that they are often based on a lack of understanding of the historical context and the persistent coloniality of global health programs in Africa (38). The content of these interventions, along with their implementation and evaluation, reveals a contextual disconnect and highlights the difficulty of breaking free from the colonial mental models in which they are rooted (39). Recognizing local knowledge, engaging with the target population to understand their motivation to change, and adapting interventions to the local context (including social norms, culture, and environmental factors) facilitates changes. However, much of the power in global health is maintained by developing a system of knowledge (worldviews, concepts, tools, methods) that is legitimized and participates in creating good food practices meanings (40). Colonial connotations continue to subtly inform, shape, and govern current nutritional standards, leaving little room for developing local responses based on community knowledge (41). The call for undoing these colonial legacies in global health (42) requires recognizing cultural and experiential knowledge (43), removing all forms of supremacy, and not disregarding Indigenous knowledge (44). Respecting multi-rationality, expertise, and ways of living (45) is an interesting starting point for redefining global health practices (46). Nutrition interventions should be considered from a decolonial perspective because food knowledge and practices hold significant cultural meaning and are deeply embedded in the symbolic universes of populations (47). The decolonial approach advanced in this paper seeks to challenge dominant Western paradigms in nutrition and public health communication by questioning the imposition of so-called “universal” solutions that often disregard local contexts (48), cultural meanings of food, and indigenous knowledge systems. It involves a critical examination of the power dynamics that shape how knowledge is produced, legitimized, and operationalized within SBCC. It advocates for centering the voices, lived experiences, and epistemologies of Senegalese communities in the design and implementation of nutrition interventions. They would include re-anchoring in populations’ territorial realities, social links, and networks. Therefore, communication for social change should involve listening to more relevant and authentic food identities for populations and promoting diverse ways of thinking about nutrition.

Proposing an alternative to globalization, the ecology of knowledge (49) defends the plurality of knowledge systems, valuing scientific, experiential, and indigenous epistemologies, as equally valid contributors to addressing public health challenges. It recognizes the intersubjective dimension, trans-scale, trans-temporal, and dynamic aspect of knowledge, an interconnected system within a human environment, emphasizing its diverse forms and interactions. This perspective emphasizes *non-relativistic dialogs among knowledge* to enable cognitive justice and dismantle monolithic Western paradigms (50). In the context of SBCC interventions, it calls for epistemic pluralism and mutual respect between global health frameworks and local knowledge traditions. Drawing on this concept, we attempt to rehabilitate territorial capabilities, which have been long obscured or delegitimized by monocultural domination (51), and aim to demonstrate that nutrition SBCC must rely on citizen knowledge rooted in everyday experience, respecting cultures and their right to define their own food and farming systems (52). Adopting such a viewpoint may require a community-and people-centered approach, one that recognizes and respects individuals’ environments, knowledge, and capacities in negotiating nutritional behaviors.

As a social practice, nutrition encompasses the habitual actions, routines, and behaviors surrounding food acquisition, preparation, and consumption that are embedded within cultural traditions, family dynamics, and social interactions (53). These practices shape how individuals and communities interact with food on a daily basis. Simultaneously, nutrition is a social construct, where the meanings and values attached to food, such as what is considered healthy or appropriate, are shaped by cultural norms, power relations, and societal discourse rather than solely by biology (54). Together, these perspectives highlight that nutrition is not merely a biological necessity but a complex social phenomenon influenced by both practiced behaviors and socially constructed meanings. So, how can we communicate ethically and responsibly to encourage stakeholders to take ownership of an intervention designed to change their nutritional behavior, which affects them? This ethical reflection invites us to consider the principles of justice (equity, sharing, and dignity), beneficence, self-determination, and humanity while balancing individual and collective responsibilities and critically examining the foundations of the social legitimacy given to recommended nutritional practices (55). With the risks of food incentives (promotion, restriction) undermining collective autonomy, it is necessary to enable individuals and groups to make their own choices by understanding the issues and risks associated with their physical, socio-economic, and lifestyle environments (56).

## Materials and methods

This article proposes a decolonial and knowledge-ecological analysis of SBCC nutrition interventions in Senegal, which involves critically examining how these programs reflect global power imbalances and epistemic hierarchies, and suggests more inclusive, culturally grounded, and environmentally aware alternatives that engage communities as co-creators, rather than passive recipients of health solutions. Specifically, we utilized the results of three surveys conducted in Senegal from 2020 to 2024, focusing on malnutrition, anemia, rice fortification programs, and culinary broths (Table 1). The National Ethics Committee for Health Research approved the protocols (SEN19/90/and 20/58).

**Table 1:** Comparative overview of three formative nutrition studies conducted in Senegal (2020–2024).

Title of the study	Period	Geographic scope	Type of study	Target groups	Unit of analysis
Study on culinary broths and their fortification	2020–2021	National (urban and rural areas)	Mixed methods (quantitative, qualitative, direct observation)	Institutional actors (regulation/control)	Individual (adult consumer or professional), household
				Producers and distributors—households—community leaders—health and consumer associations	
Ethnographic study on rice preparation and fortification (Matam)	2021–2022	Matam region (northeastern rural zone)	Mixed methods (ethnography, questionnaire, interviews, FGDs, observation)	Female heads of household	Household, school, individual (female caregiver, cook)
				School cooks	
				School staff	
				Students (via focus groups)—community leaders	
Formative research on undernutrition in children under 5 (Dakar and Saint-Louis)	2023–2024	Dakar and Saint-Louis (urban areas)	Qualitative (interviews, FGDs, SBCC mapping)	Parents (mainly mothers) of children under 5	Individual (mother/caregiver), household, community group
				Community health workers	
				Nutrition program staff	
				Community leaders	

*Study of preferences, perceptions, knowledge, attitudes, and practices related to broths in Senegal* (2020–2021): This research aimed to understand the determinants of preferences, perceptions, attitudes, and practices of stakeholders regarding culinary broths and their micronutrient fortification in Senegal. The study targeted various categories, including institutional actors involved in regulation and control, production and mass distribution, households, community leaders, and professional health and consumer associations. A mixed approach assessed people’s nutrition-related knowledge, consumer attitudes, preferences, purchase patterns, and broth demand. It explored the drivers of acceptability for fortified broths. Eight hundred eighty questionnaires, 283 interviews, and 103 focus groups were administered, with direct observations in 108 households. The results provided recommendations for developing an evidence-based approach to communication and awareness-raising on broths and their fortification.

*An ethnographic study of rice preparation, cooking methods, practices, and perceptions of rice fortification in Senegal* (2021–2022): This study aimed to analyze the acceptability of local rice fortification planned by the Senegal Committee for Micronutrient Food Fortification (COSFAM) World Food Program (WFP) in school canteens in north-east rural areas (Matam). We studied whether rice would be appropriate for micronutrient fortification and promoting nutritional practices in households and canteens. Specifically, we described stakeholders’ knowledge, attitudes, perceptions, and practices regarding rice preferences and preparation in schools and families. We focused on the preparation and cooking practices for the local rice and evaluated the social acceptability of its fortification to define an SBCC strategy. The research employed a mixed-methods approach, combining a questionnaire administered to the female head of the household with in-depth interviews, focus groups, and direct observations. Four hundred questionnaires, 128 interviews, and 47 focus groups were administered. WFP used the results to develop an SBCC strategic plan to accompany the pilot of integrating enriched rice into school feeding programs.

*Formative research on the determinants of malnutrition in children under 5 years* in Saint-Louis and Dakar (2023–2024): Using a qualitative method, we mapped nutrition SBCC initiatives, analyzed their strengths and weaknesses, and gained an understanding of the perceptions. We identified the mechanisms and causal pathways of undernutrition and documented factors at the individual, family, and community levels. We described the stakeholders’ attitudes and practices about feeding children under five, as well as anemia and malnutrition. Specific recommendations have been identified to inform ongoing nutrition strategies and adapt communication strategies for social and behavioral change, thereby improving multi-sectoral nutrition security programs.

Participants were selected using a purposive sampling strategy to ensure relevance to the research objectives: For the survey on rice fortification in Matam, inclusion criteria focused on female heads of households responsible for meal preparation, school cooks, teaching staff, and community leaders involved in school feeding programs. For the broth fortification study, we targeted adult household members who regularly use culinary broths, as well as actors involved in the production, regulation, or distribution of broths. In the formative research on child nutrition in Dakar and Saint-Louis, participants included caregivers of children under five, community health workers, nutrition program staff, and local leaders. Participants were included if they were permanent residents of the selected study areas, directly involved in household food preparation or nutrition-related decision-making, or held institutional roles in nutrition programming. Exclusion criteria applied across all studies included individuals under 18 years of age (unless involved in school-focused focus groups), temporary residents, and individuals not directly involved in food preparation, caregiving, or nutrition-related roles. The sampling approach prioritized diversity in age, occupation, and socio-economic status to capture a wide range of perspectives while maintaining relevance to the thematic focus of each study.

All three studies were conducted under our leadership, throughout the entire research cycle, from the formulation of research questions to data collection, analysis, and dissemination. Our direct involvement at every stage ensured methodological rigor, contextual sensitivity, and strong engagement with local actors and communities. This positionality also allowed for a grounded interpretation of the findings. It strengthened the reflexive, decolonial perspective that informs the present article’s critical approach to nutritional communication and behavior change strategies. Although most studies employed a mixed-methods approach, this paper uses only qualitative data to review related SBCC nutrition interventions, conducting an audit on their local adaptability and social acceptability using the Theoretical Framework of Acceptability (TFA) (57). Acceptability refers to determining how well the target population perceives and adopts an intervention, as well as how well its components meet their needs (58). It is a multifaceted construct that reflects how people delivering or receiving a healthcare intervention consider it appropriate based on anticipated or experienced cognitive and emotional responses (59). Considering that the targeted populations had some level of exposure to the SBCC nutrition interventions, we analyzed them considering user engagement, affective attitude including adherence, intentions (e.g., willingness to engage with the intervention), its actual usage, uptake (e.g., frequent interaction with the intervention) the perceived satisfaction after having engaged with the intervention; its capacity to constructively integrate sociopolitical factors, such as religion, ethics, legislation, norms. We were also interested in users’ perception of the intervention’s effectiveness, self-efficacy, opportunity costs, and willingness to recommend (60). A participatory approach has been employed during our research process to incorporate community perspectives and local priorities, yielding meaningful findings that can be translated into action or utilized to promote social change. The field research was conducted by listening to and understanding local knowledge about food and nutrition, identifying communities’ views on prioritized nutrition behaviors adapted to local contexts, and assessing what is acceptable and capable of enhancing their nutritional situation.

A thematic analysis was employed to systematically identify, organize, and interpret key patterns across the qualitative data collected through interviews, focus group discussions, and observations. Deductively, we applied a coding framework informed by critical theories of behavior change communication, decoloniality, and the ecology of knowledge. Inductively, themes also emerged from repeated close readings of field data and participant narratives (limited participation, cultural disconnect, and structural barriers). Each theme was developed based on the recurrence of ideas, values, tensions, and critiques expressed by stakeholders, such as caregivers, community leaders, and institutional actors, across the three case studies (broths, rice fortification, malnutrition) (Table 2).

**Table 2:** Thematic analysis of SBCC nutrition interventions in Senegal.

Theme	Description	Empirical illustrations	Theoretical implications
1. Limited participation	Community engagement confined to formative research, not to co-design or decision-making processes	Communities took part in FGDs and surveys but did not shape intervention strategies	Need for shared governance and transformative participation in nutrition interventions
2. Top-down communication models	Interventions are driven by national dietary guidelines and biomedical logic without context-specific adaptation	Messaging emphasized micronutrients and standardized food groups, overlooking local meanings	Demonstrates the persistence of technocratic, behaviorist paradigms
3. Cultural disconnect and epistemic gaps	Disconnection between scientific discourse and culturally grounded food beliefs and practices	Beliefs about broth-related impotence, food taboos, and gendered cooking roles excluded from official SBCC narratives	Reveals the need for epistemic justice and inclusion of plural rationalities
4. Acceptability of interventions	Acceptability is influenced by taste, trust, norms, and symbolic meanings of foods	Enriched rice poorly received in school canteens; fortified broths rejected due to myths or sensory preferences	Acceptability must be understood as socially constructed, beyond technical acceptability
5. Ethical tensions in behavior change	Imposing normative dietary messages risks undermining autonomy and cultural rights	Lack of dialog around community food choices limits ethical legitimacy of interventions	Relational ethics, informed consent, and respect for cultural worldviews
6. Structural barriers to practice change	Even when knowledge is internalized, external constraints limit its application	Families lacked resources or capacities to apply recommended feeding practices	Underscores that behavior change requires enabling environments, not just awareness
7. Participatory approach as transformative potential	True participation involves co-creation, not only consultation	Suggested local nutrition advisory groups and iterative reflection mechanisms	Aligns with decolonial frameworks emphasizing community agency and co-production
8. Ecology of knowledge	Nutrition communication must integrate multiple knowledge systems, not just biomedical science	Recognition of traditional crops, food rituals, and local heuristics alongside scientific evidence	Supports the ecology of knowledge approach: co-validity of scientific, experiential, and indigenous epistemologies
9. Toward a decolonial SBCC approach	Paradigm shift from normative, top-down messaging to pluralistic, co-constructed communication	SBCC as a space of negotiation, not transmission; centers self-determination and cultural resilience	Build inclusive, context-sensitive, and socially just nutrition interventions

This analytical strategy enabled us to trace the gap between normative public health models and lived food realities, while proposing context-sensitive alternatives grounded in community agency and plural knowledge systems.

## Results

### SBCC nutrition interventions’ adaptability and acceptability in Senegal

Through various communication techniques, SBCC aims to modify a population’s behavior by altering its knowledge, attitudes, and social norms. Within the health sector, it is a research-based, consultative, and communicative process involving dialog and partnership to create supportive environments and facilitate behavior change required to manage a public health situation (61). Its activities aim to enable changes in behavior and social norms, demanding recognition of the uniqueness and singularity of communities through interaction (62). SBCC is guided by a comprehensive ecological theory that incorporates perspectives and needs for individual-level change, as well as broader environmental and structural changes. It encompasses the need to consider people’s participation and engagement, as well as the socio-ecological context, systems, and processes that underpin health within communication initiatives.

One of the usual errors in policymaking is assuming that information and knowledge determine behavior. Such an approach postulates that if we inform people about the negative consequences of overeating or exercising too little, they will change their behavior accordingly once they have been alerted to the dangers of specific practices (63). This approach is reminiscent of a development perspective of the 1990s, which aimed to involve village communities in their development programs by mobilizing them through collectives whose actions had already been defined by technicians (64). Unfortunately, the link between knowledge, attitude, and behavior is not always direct (65), as there is a possibility that what we do is not regulated by what we think or want but influenced by our experiences, interactions, and exchanges with our environment (20). In this asymmetrical order of interaction (66), populations occupy assigned places, while professionals promulgate biomedical knowledge and undermine communities’ experience and understanding of life-world concerns (67).

Over the past few decades, the SBCC field has expanded to include more complex frameworks, such as community-based participatory approaches to design solutions for health challenges. It promotes communities’ strengths and skills, acknowledging community members as valuable contributors to the process and combining knowledge and action to improve health (68). Although new opportunities for engaging people in social change processes have opened up, there is a need to perform a thorough audit and discuss their local adaptability, social acceptability, and ethical dimensions. We focus on two significant interventions (*Fortification of local rice and culinary broths; Livestock micro-credit*) selected thanks to their SBCC components used alongside nutrition-sensitive strategies, corresponding to the CNDN’s top priorities (69).

*Fortification of local rice and culinary broths* has been increasingly recognized as a promising approach to preventing micronutrient deficiencies (70). The fortification of staple foods is one strategy WHO and the United Nations Food and Agriculture Organization (FAO) have adopted to improve dietary diversity and effectively decrease micronutrient deficiencies (71). As one recommended intervention, along with nutrition education, for scaling up in the Multisectoral Strategic Plan of Nutrition of Senegal 2017–2021 (PSMN), it consists of increasing the content of one or more micronutrients (vitamins and minerals) in food or condiments to improve their nutritional quality. In the past, women cooks used aromatic plants such as rosemary, basil, and laurel to enhance the flavor of their sauces (72). Nowadays, broths have been promoted to season dishes. Local manufacturers (INASEN, PATISEN, SENICO), competing with food industry giants (Nestle, GB Foods), are suggesting powder broth as a suitable alternative and branding it using local names: *Adja* (name), *Doli* (augment), *Jongué* (seduce). In 2009, the Senegal Bureau of Standards (ASN) made it mandatory to fortify cooking oil with vitamin A and wheat flour with iron and folic acid. COSFAM, in collaboration with WFP and Nutrition International (NI), has been exploring the expansion of large-scale food fortification programs (73). Among the most promising vehicles for fortification are broth cubes and local rice, both widely consumed in urban and rural settings (74). Although broth fortification is not new in Senegal, public awareness of this practice remains limited. Since 2013, Nestlé has fortified its Maggi cubes with iron, while GB Foods, based in Barcelona and producer of Jumbo bouillon in Africa, has incorporated vitamin A into its products. Similarly, the Senegalese company SENICO has fortified all its Kadi broth powders, available in shrimp, tomato, and chicken flavors, with vitamin A and minerals.

Local rice has also been identified as a strategic platform for fortification, particularly when integrated with social safety net programs (SSNP) such as school feeding schemes. These initiatives enable both the delivery of fortified foods to vulnerable populations and the dissemination of information on healthy diets (75). Senegalese households consume, on average, 0.24 kg of rice per person per day, making it a staple in the national diet. While around 60% of rice is still imported, mainly broken rice from India, Thailand, and Brazil (76), local production has grown significantly due to investments in modern rice mills in the Senegal River Valley, bolstered by the National Rice Self-Sufficiency Program (PNAR). To ensure the success and acceptance of fortification programs, nutritional education and behavior change campaigns have been deployed to reinforce key messages, dispel misconceptions among stakeholders and the broader public, and promote the consumption of micronutrient-rich foods. Effective communication and community awareness efforts have played a crucial role in helping consumers understand the health benefits of fortified products and increasing their willingness to incorporate them into their daily diets.

Our results suggest that while the use of bouillon cubes and other additive products has become nearly universal, this widespread adoption masks more nuanced and critical perceptions, highlighting a growing tension between the practicality and culinary appeal of broth and the health anxieties it evokes across generations and genders. As a well-flavored meal is crucial to women’s domestic work in Senegal, broth or additive products are increasingly used within households. In general, 99.5% of Senegalese households surveyed use bouillon to cook meals (77.1% use it 7 days a week). In the 24 h before the survey, 95.68% had used them to cook meals. In the southern zone, broths are consumed on average 3 days a week (20.30%), while in the southeastern zone, most households (90.60%) use them daily. However, these figures conceal disparities in attitudes to broths between generations and genders.

According to women, the broth adds flavor, color, and aroma, which is one way families appreciate the talents of a good cook. These products have become indispensable for women, who continue to use them secretly from their husbands.

The head of the household gives us our daily allowance, and we pay for the broth. He doesn't ask us to pay for it. He doesn't like the broth… When it's my turn to cook, I do everything possible to serve a pleasant meal. That's why I use the broth and hide it so that my husband doesn't see it. IDI 2_Saint-Louis Ndiébène Gandiol _Head of the kitchen

These commodities and flavor enhancers allow women to enhance their culinary practices and intensify the taste of everyday dishes (*saf sap*), a central concern of *Jongue* (77). This concept refers to seductive gestures in a woman’s everyday life, as seen in how she speaks, cooks, dresses, or presents herself in a way that makes her family happy and proud. In polygamous households, women compete to create delicious meals using different cooking “tricks” (diverse culinary additives), distinct from those prioritized by older relatives. Broth powder is preferred to add color and brightness, showing a good food taste and enhancing social prestige. Not using broth in cooking in the northern zone involves social risks for women. Even if their husbands forbid them from doing so, they do not hesitate to use broth to avoid “losing face” in front of their peers. Meals are eaten together, and this sharing exposes women to the appreciation of their peers and men. To gain social prestige, women competed with each other in ingenuity. In a restrictive environment (low fish availability and large quantities to cook), powdered broths have significantly enhanced the color and flavor of food. Although their husbands were against the broth use, they were happy when their peers praised their wives’ culinary talents. Men and grandmothers associate broths (especially Doli) with numerous pathologies such as cancer, diabetes, hypertension, stroke, and even sexual impotence:

“Doli dolii balli” (doli has crushed our bones) “doli dollat” (doli crushes the bones of the body) “safii, safroyo” (if it's tasty, you'll end up in hospital) … From the moment we started using these broths until now, there have been nothing but problems. We use these broths even when we want to sterilize cows and sheep. As a result, men now have no sexual potency, and this causes problems in couples. So, let’s get rid of the broths in Senegal because they're no good. FGD21_Tambacounda_Goudiry_Kothiary_ZR_Quartier Est_Ménage_ Male head of household

Local rice is a readily available commodity produced in villages and primarily intended for home consumption. It has become increasingly popular due to its nutritional benefits and more natural taste. In addition, because it has no added preservatives and is less broken, it contains more vitamins and has more health benefits for consumers (avoiding diabetes). While the prospect of its fortification is welcomed in school canteens, more is needed to change communities’ consumption behavior. Some respondents were skeptical that fortification has non-harmful health objectives. Fortified rice would limit children’s fertility compared to family planning and early childhood immunization.

In Fouta, people are afraid of the vitamins they give to the children because they say it's to reduce fertility. Recently, we've heard about pasteurized rice. There is flavored rice in the shops that causes diabetes, so people are suspicious of it. FGD 7_Matam_Ogo_Diandioly_Women

Households are in favor of fortifying imported rice, which is considered by many to be low in micronutrients. According to women, local rice does not need to be fortified as it contains vitamins, is more natural, has a good taste, and allows quick digestion. Some concerns are being raised about the additives, which could alter the flavor and color of local rice, leading to higher prices and potential adverse health consequences. Because its packaging and storage do not guarantee its hygiene, soaking, washing, and rinsing the rice several times helps to clean it and remove dirt, dust, and gum. However, repeating the process can cause the rice to become too soft or mushy, resulting in the loss of micronutrients. A more hygienic presentation of rice would increase consumer confidence rather than fortifying it.

Both local rice and culinary broths, despite their recognized nutritional potential, face negative perceptions regarding their fortification in Senegal. Acceptance of broth, particularly among women and adolescents, does not translate into their appreciation of its fortification and use patterns. Although these products can be identified by their packaging, women tend to interact with them less. Because broth is so controversial within households, women suggest using safe local products, such as maize, sorghum, and fonio, and milk for fortification. Several factors help explain this mistrust. First, fortified products are often associated by women with industrial transformations that are perceived as foreign, unnatural, or incompatible with traditional food practices. Women view fortification as detracting from the taste of the meal and consider that fortifying some local foods should be more tailored to their socio-economic contexts. In the case of broths, which are widely consumed yet already subject to health-related controversy, fortification is sometimes viewed as exacerbating existing risks. Men believe that fortified broth is more harmful and has adverse health effects. They suspect that rather than promoting growth, it could lead to delayed cognitive development in children, rapid fatigue, and brittle bones.

The broth is already problematic. Using it to add vitamins is tantamount to fortifying a complicated and controversial product when local products can be used. Combining vitamins with broth means creating a more harmful product, a poison, a deadly cocktail. For us, enriched broth is “doggali” (to finish) or “yok xarmi karaw rek” (to increase the hair on the sheep)—FGD21_Matam_Kanel_Ouro Sidy_Head of Household.

A lack of clear, accessible information about added micronutrients reinforces doubts, particularly among men and older individuals, who often express concerns about links to hypertension, diabetes, or sexual dysfunction. Moreover, the limited community involvement in the design and communication of fortification policies fosters a sense of exclusion, as local knowledge systems and food cultures are sidelined. These dynamics highlight the urgent need for a decolonial and culturally grounded approach to promoting fortified foods. Controversies about marketed broths have led women’s groups to encourage processing natural ingredients (*Nokoss, as natural seasoning*) to give taste to food while protecting their health*. Nokoss* is a mix of garlic, pepper, onion, chili (seeds), nététou’ (néré seeds), solu (dried and ground baobab leaves), parsley, spring onion, pepper, cloves, ginger, bay leaf, tomato, used by women to prepare natural flavor. There are also local initiatives to produce local broths, especially in Kaolack, using only natural ingredients such as dried fish, chili peppers, iodized salt, pepper, paprika, and garlic, which are mixed and presented in powder form in small sachets.

*Improving women’s incomes, food, and nutrition security through the Livestock micro-credit*: Senegal’s Nutrition Enhancement Program (PRN), launched in 2001, deployed several innovative interventions to promote key family health and nutrition behaviors, focusing on community-level interventions, community-based growth monitoring and promotion/community-based integrated management of childhood illnesses. Global attention has been paid to financial empowerment through micro-enterprise interventions and women’s livestock projects. Empowerment has been associated with various approaches that focus on access to and control over resources, including food security (78). From 2017 to 2022, the World Bank-supported initiative led by Senegal’s National Council on Nutrition and Food (CNDN) aimed to empower women at the community level in nutritionally vulnerable regions by promoting improved infant and young child feeding (IYCF) and caregiving practices. One of the project’s flagship components was a solidarity-based livestock intervention, wherein a family received a loaned animal to care for until it reproduced, thereby fostering community support and contributing to household nutrition.

I received three goats as a form of micro-credit. I had to take care of them. At the end of two years, during which I could feed them, allow them to give birth to young people, and benefit from their milk, I had to repay the loan by giving two young goats to a new beneficiary family. One condition of receiving the loan was communicating with the latest beneficiary, sharing the technical knowledge I have acquired, and raising awareness about nutrition, good hygiene practices, and the importance of goat's milk consumption by children in rural areas. IDI21_Tambacounda_Makacoulibantang_ZR_Woman, 40 years.

However, despite its promising objectives, the implementation of the program revealed a recurring and well-documented challenge: local ecological conditions, cultural norms, and community knowledge systems were insufficiently integrated into the project design. As a result, the intervention struggled to adapt to the lived realities of its beneficiaries, highlighting the ongoing need for development initiatives to move beyond top-down approaches and engage meaningfully with local contexts. Women’s economic empowerment has been a centerpiece of policy discourse in African countries (79), a means of improving household nutrition and contributing to gender equality (80). Providing livestock to increase the efficiency of community food production for both consumption and sale was assumed to modify the physical and social environment and, indirectly, the motivation to adopt the desired nutritional behavior (81). The animal is a productive asset and a resource to reinforce women’s financial autonomy and food and nutritional security. In addition to the milk the woman can give her children, the sale of goats or milk could help meet her family’s needs (73). In Senegalese rural areas, goat farming is a vital activity for women and a crucial resource for food security. Marketing of milk and its products can contribute to the fight against poverty. However, Senegalese women regret that the distributed goats did not consider local conditions:

Lending us goats is beneficial because this animal still holds high social value here and is often kept by women. We can sell, trade, or give them away to support the family. But my farm didn't survive, mainly because the borrowed goats were unsuitable. Here, we use more local Djallonké goats. This breed reproduces more efficiently throughout the year, is more adaptable to humid climatic conditions and animal diseases, and is better suited to herd grazing, which is more familiar to us here … Fatou, a 45-year-old mother of 7, Goudiry.

The West African dwarf goat, also known as the Djallonké, is notable for its early reproductive maturity, high fertility, and strong adaptability to tropical climates. It is especially valued for its nutrient-rich milk, which plays a key role in local diets and is often used in the production of traditional cheeses. Moreover, its natural resistance to diseases such as trypanosomiasis makes it a critical asset for rural households (82). However, attempts to introduce non-native goat breeds poorly suited to local ecological conditions have led to significant livestock losses. These failures have had a disproportionate impact on women, who are often the primary caretakers and beneficiaries of household livestock. In some cases, such losses have increased women’s vulnerability and reliance on food aid programs, such as WFP voucher schemes.

With the poor rainfall of last winter’s campaign, I could not cultivate the previous year's crops, and the goats suffered from a lack of fodder. I was living under tremendous stress and was contemplating selling my goats even more to feed them. During the fall, I depended on my two goats and market gardening to meet my family's nutritional needs. » D, Female, Dodel, female head of household.

Even though goat rearing is more accessible for women, climate change has limited their access to pastures and other resources. They cannot leave the farm to graze the goats, which do not adapt well to pastures or obtain sufficient quantities of fodder without human help. Moreover, in Senegal, women could own animals and build up a small herd, but the livestock-raising activity did not improve their financial and nutritional capacities. The consumption of goat products is primarily shaped by access to affordable food options, as well as perceptions and beliefs (83). In pastoral Fulani communities, the milk produced is not necessarily intended for family consumption, and the money earned from its sale sometimes encourages women to buy imported rice. Goat’s milk consumption is instead replaced with skimmed milk powder imported from Europe, sold in sachets in shops or on weekly market days (84). Within the households targeted by the goat-transfer intervention, awareness-raising and nutrition education activities were primarily directed toward the women recipients of the animals. While this focus aimed to empower women as key actors in child nutrition, it inadvertently sidelined other influential household members—particularly husbands and grandmothers—who play crucial roles in decision-making around food, caregiving, and household resource allocation. In many Senegalese households, grandmothers are custodians of culinary knowledge and caregiving norms, while men often control financial resources and livestock. Men emphasized that they had received little information on child nutrition issues due to communication that was primarily aimed at women. Microcredit projects showed mixed effects, without considering categories that support women’s involvement, as the loans taken were hardly reimbursed. Some women felt unable to make nutritional changes without the support of their male partners, as men held some power over household nutrition because of the financial assistance they provided.

Men have been sidelined, as if women alone could solve the problems of the family. As much as we need our husbands, they need us. If my husband had been involved in this goat project, he would have received information and supported me in feeding and caring for the animals. In the family, we can't consider and promote women without the support of men. That's our reality. Moreover, times have changed, and an increasing number of men are interested in their wives' well-being. Sometimes, when I take a loan to improve my family's situation, it is thanks to my husband, who supports me in repaying the loan. I am a 37-year-old woman, Beneficiary of a goat loan in Goudiry, Tambacounda.

Excluding these actors from awareness activities limited the effectiveness of the intervention, as it failed to foster a shared understanding or collective responsibility for improving household nutrition. This gap reflects a common pitfall in gender-targeted programming, where women’s empowerment is pursued without sufficiently engaging the broader social ecosystem that shapes their agency and capacity to act. Although the woman-centered approach can allow women to take control of their agency, culturally biased attitudes should be addressed to enable women to exploit their potential at the family, community, and national levels (85). The intervention’s limited adaptability to specific ecological, social, and gender dynamics underscores a broader issue in development programming, where technocratic solutions, even when well-intentioned, may falter without genuine engagement with local realities and the co-construction of strategies with communities themselves. This reflects broader critiques in global nutrition programming, where externally driven solutions often prioritize biomedical or economic rationales over social, cultural, and gendered dynamics (86). Recent research emphasizes that sustainable nutrition outcomes require co-designed interventions that are not only technically sound but also socially embedded and culturally resonant (87). Without meaningful engagement with communities and respect for local practices, such initiatives risk undermining the very empowerment they seek to promote (88).

### What can we learn from SBCC nutrition practices in Senegal? A decolonial analysis

The various Senegalese initiatives discussed rely on community-level interventions, often supplemented by social and behavior change communication (SBCC) strategies aimed at improving nutrition-related practices at the household and community levels. While these efforts aim to inform and persuade individuals to adopt specific behaviors or products, they often operate within a technocratic framework that prioritizes externally defined knowledge, overlooking the complex social, cultural, and political realities that shape dietary behaviors. Such approaches tend to reproduce colonial legacies by marginalizing local knowledge systems, disregarding community voices, and imposing normative models of “correct” nutrition that do not resonate with lived experiences (89). Despite their diversity, these interventions have often been perceived as poorly adapted and only partially accepted by the communities, which limits their effectiveness and sustainability.

*Prioritized messages and nutritional standards are less sensitive to the ecology of knowledge*. Most nutrition SBCC interventions are inspired by the National Food Recommendations (NFRs), which are guidelines established by countries *to encourag*e the population to make healthier food choices, such as those rich in micronutrients. Such an approach perpetuates the homogenization of knowledge production by exporting Western-inspired notions of *good* or *desirable* food practices. The transfer model that underpins these interventions presupposes a *deficit,* thus replicating colonial power hierarchies that portray the South as the location of problems and backwardness and the North as the location of solutions and progress (90). Historically, knowledge of health communication campaigns emerged from within a racist ontology that constructed the dark-skinned masses of the Global South as populations to be controlled, shaping the categories of knowledge, attitude, and behavior as the basis for configuring the pathways for change (38). National institutions promote nutrition and food solutions and practices that are structurally dependent on scientific instruments guiding their vision of risks, and good nutrition practices (91).

*Tensions Between Global Nutrition Expertise and Local Practices in Senegal*: In Senegal, the development of the RANs was led by the MoHSA, the CNDN, and international experts. FAO supported the country in developing and implementing Food-Based Dietary Guidelines (FBDG), by providing external partners bringing expertise, resources, and management for local interventions, with often a gap in understanding relevant local shared values (92). International experts have typically sought to transform individuals’ knowledge, beliefs, and capacities to act by selecting key behaviors, standards, and practice guidelines in advance and then disseminating simple messages to audiences through multiple channels, such as interpersonal communication, health workers, and the media. Exclusive breastfeeding serves as a particularly revealing case of how expert-driven nutrition knowledge can become detached from the social and cultural contexts in which maternal and infant care practices unfold. In Senegal, decisions about infant feeding are rarely made by mothers alone. Older women, especially grandmothers, often hold considerable authority in matters of childbirth, breastfeeding, and early childcare (93). Yet many global health interventions promoting exclusive breastfeeding tend to target individual mothers with simplified messages, ignoring the influential roles of extended family and community actors. These deeply rooted meanings often conflict with biomedical prescriptions, rendering externally promoted standards less acceptable or even incomprehensible in specific settings.

The push for behavior change through standardized “best practices” also reflects broader structural dynamics, including institutional funding priorities, segmentation within the agri-food industry, and risk assessments shaped by international expertise. When global norms clash with diverse local realities, they often lead to implementation gaps, resistance, and unintended consequences, raising questions about the cultural relevance and legitimacy of mainstream nutrition interventions. In Senegal, some communities resist fortified broths not only for their perceived artificiality or “modern” taste, but also due to rumors and cultural myths, such as the belief that these products cause male impotence. This belief, although unsubstantiated by evidence, plays a significant role in shaping consumption patterns, particularly among men who typically make decisions about food. Scientific nutrition campaigns may label such beliefs as misinformation, but doing so without cultural sensitivity can generate mistrust, resistance, or accusations of cultural imperialism. The trade-off is not between culture vs. science, but between imposition vs. integration. Public health must strive to bridge worldviews by validating local knowledge wherever possible, challenging harmful myths through dialog rather than dismissal, and grounding interventions in scientific evidence that resonates with local values and perspectives.

Targeting women’s empowerment through microcredit and nutrition education is rooted in a philosophical vision that prioritizes the perspectives of the oppressed and women, aiming to raise their awareness of the relations of domination that marginalize them. Empowerment should allow them to transform unequal economic, political, and social structures so that they could express themselves and overcome the dominance to which they were subject (94). With the adoption of international development institutions, empowerment has gradually become synonymous with individual capacity, achievement, and maximizing individual interests. It has been used to legitimize existing top-down policies and programs (95). Such an approach embeds global North values of personal autonomy as universal (96), disregarding some local values related to family and collective decision-making, which are core to many African communities. This illustrates how coloniality in knowledge production informs dominant approaches to shifting social norms. Focusing exclusively on women did not consider *the roles, rights, responsibilities, and power relations associated with being male or female* and how power dynamics within households may limit women’s ability to adopt healthy behaviors and access nutritious foods. These empowerment programs also assume that grandmothers primarily influence women negatively, demonstrating a limited understanding of cultural contexts where seniority confers authority on female elders about infant health and nutrition. Excluding men from the nutrition discussions and activities reinforced existing gender divisions and the notion of child feeding and nutrition as women’s business (97). Furthermore, the social entrepreneurship environment for women is inextricably linked to a family dimension that affects their ability to maintain their business.

*Overlooking food ecology: neglected impact of climate* var*iability and ecosystem changes on Nutrition in Senegal’s SBCC Interventions*: Climate variability and environmental degradation are escalating threats to local food systems in Senegal and the broader Sahel region, significantly affecting agricultural productivity, food availability, and dietary diversity (98). The intersection of climate change, food systems, and nutrition is a critical area of study in Senegal, as the country’s diverse agroecological zones are increasingly affected by shifting rainfall patterns, droughts, and rising temperatures. These ecological shifts are deeply intertwined with nutrition outcomes, as fluctuations in crop yields and access to diverse foods directly contribute to malnutrition, particularly among vulnerable groups such as young children and women of reproductive age (99). Emerging research underscores that climate variability and ecosystem changes have profound implications for local food security and nutritional status (100). Therefore, nutrition interventions, including SBCC programs, must intentionally incorporate climate adaptation strategies and ecological sustainability principles to enhance their long-term effectiveness and resilience. Yet, many SBCC campaigns remain detached from these realities, focusing narrowly on individual behaviors and biomedical recommendations (e.g., iron-rich diets, exclusive breastfeeding, fortified foods), while overlooking the structural and ecological constraints that hinder their feasibility. This results in misaligned messages, particularly when promoted foods (e.g., imported fortified rice or broth cubes) are unaffordable, unavailable, or culturally displaced by climate-driven changes in local food practices (101). Understanding these ecological dynamics is crucial for designing resilient, context-sensitive nutrition interventions that support sustainable food systems and protect community health in the face of ongoing environmental challenges. By neglecting the integration of food ecology and climate considerations, current interventions ended up being disconnected from the lived realities of populations whose nutritional well-being is directly shaped by ecological transformations. In Senegal, empirical study advocate for nutrition interventions that are climate-sensitive and community-engaged to address evolving local challenges (102).

*The lack of participatory approaches does not facilitate culturally nuanced and more readily accepted behavior change programs*: In Senegal, health promoters disseminate interventions mainly based on health belief models, knowledge, and attitude. Even if communities are incorporated or consulted in this process, they are denied the capacity to develop theories, own them, and mobilize through them toward transforming the meaning of nutrition standards. Sometimes, participation is limited to simply co-opting people as amateur observers or passive recorders to provide data to experts, with no direct benefits to themselves, without bending scientific concerns to local needs (103). Social mobilizers offer information and support to communities and then engage in strategic dialogs to integrate the promoted knowledge. Usually, qualitative methods like household surveys, focus group discussions, and key-informant interviews are considered “participatory” by policymakers on their own; however, they are not, as these interactions are often one-directional and not design-oriented (104). While logic models, formative qualitative research, and case–control studies are valuable tools, relying solely on them can limit program development, as they often lack a comprehensive understanding of behavior change strategies and audience context (105). A participatory SBCC nutrition intervention acknowledges collaboration with communities without assumptions about their held values (106). It involves actively engaging communities and individuals in designing, implementing, and evaluating nutrition programs to ensure they are culturally relevant and address local needs, ultimately leading to more sustainable and effective behavior change. It envisions essential stakeholders as co-creators and co-owners of the final intervention. This approach enables context-appropriate nutrition-focused SBCC, enhancing awareness creation, community empowerment, and ownership (107).

*Existing SBCC nutrition programs have done little to build or sustain the types of capacities that are critical for durable behavior change*, particularly perceived self-efficacy, confidence, and intrinsic motivation. While these interventions have increased awareness of nutrition and hygiene practices, they tend to target behavior change at the individual or household level, often through didactic messaging. This narrow focus neglects the importance of collective agency and the enabling social environments necessary for sustained adoption of new practices. Moreover, insufficient investment in developing shared capabilities, community norms, and collective efficacy limits the reach and impact of these programs. Without engaging communities as active agents, rather than passive recipients of messages, SBCC strategies risk remaining superficial and ineffective, especially in settings where structural barriers and social norms significantly influence health and nutrition behaviors, as much as knowledge alone (108). Instead of overlooking and excluding men from gender and empowerment policy design, involving them in nutrition and caring for pregnant women and children could help to re-equilibrate roles within couples and contribute to supporting women’s empowerment and gender equality. Interventions to increase male involvement in maternal and newborn health have also been linked to increased couple communication and equitable decision-making, contributing to improved health and care-seeking outcomes (109). To mobilize this target group in malnutrition awareness-raising activities, it is essential to use existing platforms that already engage them, such as Husbands’ Schools (110), and integrate nutrition into these activities or schedule home visits when men are home. Other gender-transformative models used elsewhere involve care groups invited to join men’s groups to discuss gender equity, nutrition, and health topics in a safe space, reinforcing desired behaviors.

*Changing social norms to bring behavioral change also raises an ethical dimension that is often overlooked but crucial to the appropriateness and effectiveness of SBCC interventions*. Ethical principles for SBCC include emphasizing the importance of citizen agency, respecting diversity and culture, and a commitment to participation through dialog (111). However, the loud noise about specific nutrition standards overlooks local knowledge, rooted in social realities, and fails to promote intercultural dialog or human rights. The interventions we analyzed denied local communities self-determination, social intelligence, and the ability to hear their voices and perspectives regarding good food practices. Since the nutritional norms correlate with cultural and traditional aspects, changing them requires negotiating communities’ rights, priorities, and “social food space.” In Senegal, behavior change intervention combined peer-to-peer learning sessions with media and mobile phone audio messages promoting diverse prioritized nutrition behaviors, including diet diversity and consumption. They encouraged a change in individuals’ intentions and decision-making via indirect proposition and reinforcement. Throughout the programs, listeners were encouraged to call in to ask the community influencers questions on the discussed topics and how to apply the lessons practically. Community voices are placed at the center, providing nutrition-related messages whose content has been defined by external funders. Nutrition, Learning, Rehabilitation, and Awakening Centers (FARNE) have been established in rural areas, with activities focused on restoring nutritional status (curative dimension) and sharing knowledge with mothers to promote behavioral change. The main activities focus on regular weighing to monitor the child’s nutritional status, engaging model mothers in sharing their experience and know-how, raising awareness performing, dramas using songs and poems to convey key messages based on the prioritized behaviors: breastfeeding, hygiene, and sanitation, food groups, gradual weaning, malaria, diarrhea, etc. However, while this model aims to respect a form of free will by working on the architecture of choices, its practice can amount to manipulation, influence, or discreet pressure on a person to decide when he does not want to. It contradicts the value of autonomous individual choice. The women who stopped using FARNE’s services complained about the lack of recognition as mothers who care and have knowledge about their children’s health. Instead of listening to their needs and the resources available, they felt pressured to adopt new behaviors. Most of these media campaign activities were based on nudge and behavioral economics techniques, which aimed to predict people’s decisions and behaviors while respecting their freedom of choice. Nudges inspired by behavioral economics and neuroscience (112) have emerged, seeking to initiate behavior change, not by suppressing those deemed less healthy but by supporting choices for healthier solutions. Individuals can also be “accompanied” by raising their awareness of social norms and receiving feedback on their behavior. However, the promoted messages resembled a “kick” to force individuals to behave in a way that goes against their will. Motivation to change should not depend on pressure or fear, but rather on the individual’s and group’s ability to appreciate and find interest in the promoted behavior. Changing nutritional behavior may not have the desired effect if attitudes are manipulated through persuasion without considering the target communities’ geographical, cultural, and social contexts and realities, which significantly influence family practices. Social-ecological models have helped us understand that behavior is multifaceted, with social and environmental issues being important contributing factors (113). Rather than deciphering how extraterritorial models are appropriated within a national territory, we need to focus on how to promote local food habits promoted, transformed, or adapted, focusing on returning and giving space to cultural heritage and traditional knowledge, recognizing the value of this heritage for contemporary practices and group identity, and fostering respect (114). SBCC must be an intercultural space that negotiates ways of being in relation (115).

## Discussion

Despite the widespread adoption of Social and Behavior Change Communication (SBCC) strategies in nutrition programming across Senegal, their effectiveness remains limited when they fail to build community capacity or meaningfully engage with the broader sociocultural context. This analysis reveals that evidence of sustained and transformative change in household food practices is still scarce. These findings underscore the pressing need to reorient SBCC interventions toward decolonial, participatory frameworks that prioritize local epistemologies, address structural inequities, and support the co-creation of nutrition solutions rooted in the lived realities and values of the communities they intend to serve.

*Beyond biomedicine: integrating local food knowledge in SBCC interventions*: In the SBCC interventions examined, Western biomedical models of nutrition significantly influence both the content and framing of health messages, with limited consideration for local knowledge systems. This results in a pronounced epistemological gap between the scientific logic of nutrition, centered on calories, micronutrients, and biochemical processes, and the cultural, symbolic, and experiential frameworks through which communities interpret food. These include vernacular understandings of food “warmth” or “coolness, “beliefs tied to spiritual purity, sensory memory, and deeply gendered practices of food preparation. Such disjunction can hinder message uptake, reduce trust, and ultimately limit the effectiveness of interventions aimed at improving dietary practices. Recent discussions in global health research regarding neo-colonialism have emphasized the need to design better interventions that incorporate relevant elements of local culture (116). Calls have been made to open up behavior change policy-making to more diverse knowledge perspectives, requiring an ethic of knowledge coexistence and complementarity (117). Behavior change involves consideration of ability, motivation, and dietary knowledge that incorporates cultural food preferences, addresses motivational issues and their belief in the health benefits of dietary change, and accommodates environmental factors (social influences, local food, financial constraints) (118). A recent study grounded in the ecology of knowledge approach emphasizes that building truly inclusive and sustainable food systems requires rethinking both research and policy through a decolonial lens (119). This involves centering indigenous priorities, knowledge systems, and governance structures in the design and implementation of interventions. Drawing on action-research projects with communities in China (Naxi and Yi), Kenya (Mijikenda), India (Lepcha), and Peru (Quechua), the study highlights how co-producing interventions with community researchers, focused on traditional crops, rituals, and ecological practices, can strengthen food security and resilience. By promoting the co-management of knowledge between scientific and Indigenous epistemologies, the study offers a concrete example of how an ecology of knowledge framework can be applied in nutrition and food systems research. Interventions should avoid top-down, expert-driven models that reinforce the superiority of Western nutrition science. Instead, they should operationalize the ecology of knowledge by validating diverse systems of knowing and enabling shared decision-making in the production of knowledge.

*Co-creating change: Transforming SBCC through indigenous knowledge, social norms, and participatory practice*: In Senegal, the SBCC interventions focus heavily on raising individual awareness and knowledge, yet they insufficiently invest in fostering collective efficacy, reshaping community norms, and enabling social support mechanisms that are critical for sustainable behavior change. This narrow individualistic approach risks overlooking the complex interplay of social, cultural, and structural determinants that shape nutrition-related behaviors, such as gender roles, economic constraints, and traditional caregiving hierarchies. The community-based nutrition interventions we analyzed lack an understanding of and consideration for gendered household dynamics and ecological perspectives in behavior change promotion (120). These shortcomings contribute to persistent implementation gaps, limiting the long-term impact of nutrition interventions. SBCC programs can shift norms at the cognitive level, but achieving sustained behavioral change requires addressing collective, structural and cultural realities. Interventions need to go beyond individual attitudes to challenge collective norms, through community-wide dialog, role modeling, or gender-transformative programming. In Ethiopia, SBCC targeting both mothers and fathers, linked with food vouchers, examined paternal involvement in complementary feeding (121). Fathers in the BCC group adopted more gender-equal beliefs and expressed more supportive attitudes about being involved in feeding and childcare after the SBCC intervention; however, these shifts in attitude were not consistently translated into concrete actions (e.g., helping with feeding or food preparation). These findings echo growing calls for more participatory and systems-oriented approaches that move beyond behaviorist models to meaningfully engage with the lived experiences, social norms, and power dynamics that underpin nutrition outcomes. Embracing this paradigm requires decolonizing both the content and the processes of nutrition programming, centering indigenous and community-based knowledge, and resisting the universalization of Western nutritional norms. Rather than framing communities as passive recipients of knowledge, it is essential to recognize them as co-creators of solutions, whose cultural practices, structural constraints, and epistemologies must inform every stage of intervention design and implementation. Learning from local culture, history, and indigenous food knowledge can enhance the effectiveness of behavioral change (122). An iterative engagement process with communities should provide more significant opportunities to improve nutrition and change behavior, be culturally sensitive, address broader sociocultural standards, and consider social norms, socioeconomic influences, migration history, family structures, and roles (123). Culturally sensitive nutrition income-generating activities should empower women while encouraging men’s involvement to build fairer and more supportive relationships within households. For this reason, *community knowledge*, as the body of knowledge practices, innovations, or technologies, often built through local communities lived experiences, cultural practices, and social interactions (124) must be at the heart of communication practices.

Designing behavior change interventions requires a community-oriented and human-centered approach, engaging with the target population, understanding their motivation to change, empowering their capacities, and adapting interventions to the contexts that facilitate change, including the environment and social networks (125). Only through such holistic and context-sensitive frameworks can equity gaps be reduced and sustainable improvements in nutrition and health outcomes be achieved. In India, a recent study has shown that participation in cash transfers and food rations further improved maternal diet quality, especially when combined with SBCC. However, improvements in child diets were less consistent, suggesting barriers to intra-household food allocation or feeding practices (126). Lower diet quality remained among poorer households despite program exposure, indicating that SBCC alone may be insufficient without addressing underlying poverty and access constraints. Focus on children’s diets must go beyond household food availability, addressing caregiver practices, feeding knowledge, and gender dynamics. The study emphasizes the importance of holistic, integrated strategies that address both knowledge and access, particularly among vulnerable populations.

*Centering Local contexts, voices and values in participatory nutrition interventions*: Participatory approaches have gained broad interest as a vehicle for allowing participants’ experiences and voices to inform action (127). Formative research, including interviews, focus groups, or direct observations conducted at the beginning of SBCC programs, has been considered essential for enabling community collaboration. Although these methods can effectively inform intervention designs, they lack an understanding of locally relevant cultural elements that can be easily incorporated into intervention design (128). When supported by co-creation approaches, formative research can provide more culturally contextualized SBCC for improving nutrition and social protection delivery to vulnerable groups (129). One promising approach is the *WeValue InSitu* (130), developed from culturally informed protocols. It empowers local groups to express their shared values, which can then be transformed into tools for informed decision-making and the development of new policies. A recent stunting intervention (92) engaged stakeholder groups in exploring relevant local life practices and cultural elements related to child stunting. Themes found appropriate for locally acceptable food interventions (gender roles, social hierarchies, health service access challenges, traditional beliefs) were used for designing the protocol. Although this approach involved communities’ perspectives on co-design, our results advocate prioritizing the co-creation process, which requires collaboration and delegated power beyond the design phase to include implementation, evaluation, and decision-making. SBCC nutrition interventions must open more spaces to ensure community voices are heard and achieve sustainable, inclusive participation. The participatory approach is a collaborative and iterative design that focuses on reflecting with and planning alongside local people, rather than for them (131). It involves meaningful co-creation activities at the community level, giving key stakeholders more liberty to shape the process flow and co-create the interventions directly. Values must inform this bottom-up collaboration process, including diversity, mutual trust, openness, autonomy, freedom, respect, shared expertise, responsibility, and effective decision-making (132). SBCC interventions require emphasizing a collaborative-based social action process: rather than offering solutions, health promoters must tap into what the communities know and recognize as their strengths, partner with them to identify problems and their solutions, make decisions, and implement them. Understanding how society constructs and represents food and nutrition is fundamental to engaging in dialog about co-creating appropriate practices at a given time (133). *T*o promote nutritional and behavioral change, the prioritized standards and good food practices must be adapted to the needs of local populations and *oriented toward localized life projects and life spaces* (134). Communication interventions aimed at changing people’s eating behaviors call into question the lives, capacities, and beliefs of societies, thus raising ethical concerns: motivating people to change how they eat and feed themselves cannot be done without intruding into their lives (135). A fundamental shift is fostering participatory dialog, which requires the decentralization of egos and making concessions to promote mutual understanding and consensus (136).

*Toward a climate-Responsive SBCC: reintegrating ecological knowledge into nutrition Interventions in Senegal*: Nutrition and SBCC programs must embed food ecology and climate adaptation into their design, ensuring that both expertise and access are addressed in tandem. Climate change is already reshaping food ecologies across the Sahel, including Senegal. Erratic rainfall, soil degradation, increased temperatures, and more frequent climate shocks (e.g., droughts and floods) disrupt agricultural calendars, reduce yields, and limit the availability of diverse and nutritious food crops, mainly traditional staples such as millet, sorghum, and leafy greens (137). These ecological pressures directly affect household food security, dietary diversity, and the availability of micronutrient-rich foods, which are core targets of SBCC nutrition messages (138). Despite growing awareness of the interdependence between environmental health and human nutrition, SBCC interventions in Senegal often fail to account for climate variability and ecosystem degradation that critically shape local food systems. Strategies should promote resilient local food systems, elevate traditional ecological knowledge, and support community-based adaptation efforts that help populations navigate growing climate uncertainties. This is particularly urgent for vulnerable populations such as women and children, whose dietary needs are disproportionately affected by both social and ecological stresses. Furthermore, there is limited integration of indigenous environmental knowledge and community-based climate adaptation strategies into SBCC frameworks. Communities often possess valuable insights into seasonality, food preservation, and resilient cropping systems, yet these are underutilized in intervention design. A decolonial, ecology-informed SBCC approach would actively incorporate such knowledge, making interventions more context-sensitive and resilient to environmental uncertainty (139).

*Recommendations*: While this paper critiques the dominance of top-down, behaviorist SBCC models and the marginalization of local knowledge systems, it does not stop at critique. Instead, we propose actionable pathways rooted in the lived experiences of Senegalese communities to reconfigure SBCC interventions through:

- Participatory approaches that center community voices in both design and implementation.
- Integration of ecological knowledge, including food systems impacted by climate variability;
- Institutional recommendations for aligning SBCC strategies with agroecological and sociocultural realities;
- Ethical and gender-sensitive engagement, recognizing community agency and autonomy.

To enhance the cultural relevance and effectiveness of SBCC nutrition interventions, we recommend adopting a decolonial and ecology of knowledge approach. This entails integrating local food beliefs and practices into formative research, co-creating messages with community knowledge holders, and using culturally embedded communication tools such as storytelling, music, and ritual idioms. SBCC interventions should begin with participatory ethnographic research that documents community-based food beliefs, preparation practices, taboos, and healing systems. This ensures that local epistemologies are treated not as peripheral, but as central inputs in the design of interventions. Rehabilitate “hidden knowledges,” e.g., indigenous fermentation, complementary food preservation, food taboos protecting against disease. Nutrition messages should support and valorize locally available, culturally significant foods (e.g., indigenous grains, medicinal herbs), and resist overly prescriptive dietary guidelines that depend on imported or inaccessible products. Health communication should be co-designed with elders, traditional healers, women caregivers, and other cultural authorities. This collaborative process enhances cultural acceptability and enables hybrid knowledge production that bridges biomedical concepts with lived experiences of food. SBCC nutrition interventions should strive to be culturally compelling by building on existing knowledge, practices, skills, and priorities, engaging and co-leading with local communities, and nestling within social and ecological landscapes (140). Establishing this partnership requires promoting *social participation,* which implies a reciprocal, egalitarian exchange between the individual and the people with whom they interact in their life contexts. It means participating in collective action, joining forces to identify priorities, undertaking projects, making decisions, and issuing demands (141). This requires collective and public awareness of one’s ability to recognize a problematic situation, attribute its causes and responsibilities, and anticipate its consequences while understanding the threat of power imbalance that often looms over practitioners (142). Therefore, it is essential to ensure that feedback from communities is respected and that no decision is made without being co-created with them.

Moreover, interventions should align with local food systems and promote biocultural heritage, while establishing community-led feedback mechanisms. To address underlying power dynamics and structural barriers, SBCC nutrition interventions should actively strengthen community ownership and agency, by incorporating structured mechanisms for community reflection and feedback. This enables participants to critically engage with, question, and revise SBCC content and delivery methods. Regular community dialog sessions should be scheduled at key implementation stages to ensure strategies remain grounded in lived experiences and evolving local realities. A typical community engagement strategy should focus on establishing a Community Advisory Board (CAB). However, they tend to operate on the “involve” level, with community members providing feedback throughout the research process. A key point is to incorporate these actors’ input and collaboration throughout the process to achieve a knowledge-oriented co-production or to demonstrate sensitivity to their needs and concerns (143). CABs also need to operate at the levels of “collaborate” and “empower” within the participation spectrum (144). This means going beyond merely informing or consulting communities and instead fostering deep, sustained engagement. At the “collaborate” level, community members and program leaders work together in every phase of an intervention—from design and planning to implementation and evaluation. Shared decision-making is central: community voices are not only heard but actively shape outcomes. At the “empower” level, communities are given genuine authority and leadership in the process. This involves co-leadership structures where community representatives have equal footing with external actors, can set agendas, and hold decision-making power. Operating at these levels requires creating spaces for continuous dialog, active listening, and mutual respect. It also means prioritizing transparency and trust, recognizing local knowledge as equal to expert knowledge, and ensuring that interventions reflect community priorities, not just institutional goals. In doing so, CABs become vehicles for ethical, inclusive, and culturally grounded public health action (145). These strategies can reduce epistemological asymmetries, foster community ownership, and improve the sustainability and legitimacy of SBCC programs.

### Limitations

While this study draws on in-depth research conducted in specific regions, namely Matam in the northeast and Dakar and Saint-Louis in the urban coastal zone, its findings may not be fully generalizable to the entire country. Senegal is marked by significant regional diversity in terms of socio-cultural norms, dietary practices, livelihood systems, and access to health and nutrition services. As such, the perceptions, behaviors, and structural constraints identified in these study sites may differ from those in other areas, particularly in southern or central regions with distinct ethnic, linguistic, or ecological contexts. This geographic limitation underscores the need for locally grounded, region-specific research and the importance of avoiding one-size-fits-all approaches in the design of SBCC interventions. Future studies should expand to additional regions to capture a broader spectrum of food cultures and community dynamics across Senegal.

## Conclusion

This paper has examined critical tensions in health promotion and global nutrition interventions that often rely on theoretical frameworks and behavior change models rooted in Western epistemologies and transferred into African contexts, including Senegal. A decolonial and culturally grounded perspective is essential for making such interventions effective, ethical, and locally meaningful. Nutrition education and behavior change strategies must go beyond the mere transmission of biomedical information. Effective communication requires dialog, negotiation, and co-construction with communities, anchored in their lived experiences, constraints, and social realities.

Behavior change can only occur when communities recognize the relevance of proposed actions, understand the perceived risks, and actively participate in defining both the problems and the solutions. Interventions must respect community members’ rights to choose, acknowledge their socio-economic conditions, and aim to meet their self-identified needs. Rather than seeking to erase local norms and practices, SBCC initiatives should engage with them critically, recognizing that some cultural norms can support improved nutritional outcomes. Understanding these dynamics can inform the promotion of food value chains that are not only nutritionally sound but also culturally and socially acceptable, especially for women and children.

Addressing nutritional challenges demands genuine community collaboration and partnership. Experts should serve as facilitators, supporting communities in identifying, articulating, and addressing their own nutrition concerns. This requires a commitment to community-led transformation, grounded in mutual learning, trust-building, and respect for local knowledge systems. Nutrition interventions must adopt an ethical, gender-sensitive, and historically informed approach that fosters community agency and resilience. Such an approach opens space for plural ways of knowing and supports transformative learning, where communities are not passive recipients but co-creators of solutions.

Ultimately, SBCC nutrition interventions must be reimagined as inclusive, dialogical processes that draw on the intellectual contributions of Southern knowledge holders. This shift is crucial to building more just, context-sensitive, and sustainable responses to the complex food and nutrition challenges facing the Global South (146).

## Data Availability

The raw data supporting the conclusions of this article will be made available by the authors, without undue reservation.
